# Case report: Pathological complete response induced by immunochemotherapy in a case of Pulmonary Sarcomatoid Carcinoma staged IIIA-N2

**DOI:** 10.3389/fimmu.2024.1374270

**Published:** 2024-04-08

**Authors:** Yishu Guo, Xianling Liu, Hao Tang, Zhenhua Qiu, Fang Ma, Ao’ran Hu, Chaoyuan Liu, Yapeng Wang

**Affiliations:** ^1^Department of Oncology, The Second Xiangya Hospital, Central South University, Changsha, China; ^2^Department of Cardio-thoracic Surgery, The Third Xiangya Hospital, Changsha, China

**Keywords:** pulmonary sarcomatoid carcinoma, immunotherapy, immunochemotherapy, neoadjuvant therapy, conversional therapy, pathological complete response

## Abstract

Pulmonary sarcomatoid carcinoma (PSC) represents a rare and highly aggressive variant of lung cancer, characterized by its recalcitrance to conventional therapeutic modalities and the attendant dismal prognosis it confers. Recent breakthroughs in immunotherapy have presented novel prospects for PSC patients; nevertheless, the utility of neoadjuvant/conversional immunotherapy in the context of PSC remains ambiguous. In this report, we present a middle-aged male presenting with Stage III PSC, notable for its high expression of the programmed death-ligand 1 (PD-L1), initially deemed as non-resectable for sizeable tumor mass and multiple lymph nodes metastases. The patient underwent a transformation to a resectable state after a regimen of three cycles of platinum-based chemotherapy plus immunotherapy. Following definitive surgical resection, the individual realized a pathological complete response (pCR), culminating in a significant prolongation of event-free survival (EFS). This case underscores the viability of employing immunochemotherapy as a neoadjuvant/conversional strategy for chosen cases of PSC.

## Introduction

Pulmonary sarcomatoid carcinoma (PSC) constitutes a rare variant within the spectrum of lung malignancies, representing a mere 0.1-0.4% of all non-small cell lung cancer (NSCLC) cases (1, 2). PSC is marked by an extraordinary aggressive behavior, yet it evades standardized therapeutic approaches, owing to its inherent resistance to conventional interventions including radiotherapy, chemotherapy, and targeted regimens. Those with metastatic disease treated with platinum-based chemotherapy have a median overall survival time (OS) of approximately 7 months, which is poor when compared to other non-small cell lung cancers (3).

Immune checkpoint inhibitors (ICIs) based immunotherapy has orchestrated a transformative paradigm shift in cancer therapeutics, encompassing the neoadjuvant domain as well. Recent reports have highlighted the favorable response of PSC to immunochemotherapy; however, investigations specific to the neoadjuvant or conversional contexts remain absent. In this context, we present an exceptional case of locally advanced, unresectable PSC, which underwent successful conversion to a resectable state, achieving a complete pathological response following immunochemotherapy, and sustaining an extensive Event-Free Survival (EFS) exceeding 32 months.

### Patient information

**Figure 1:**
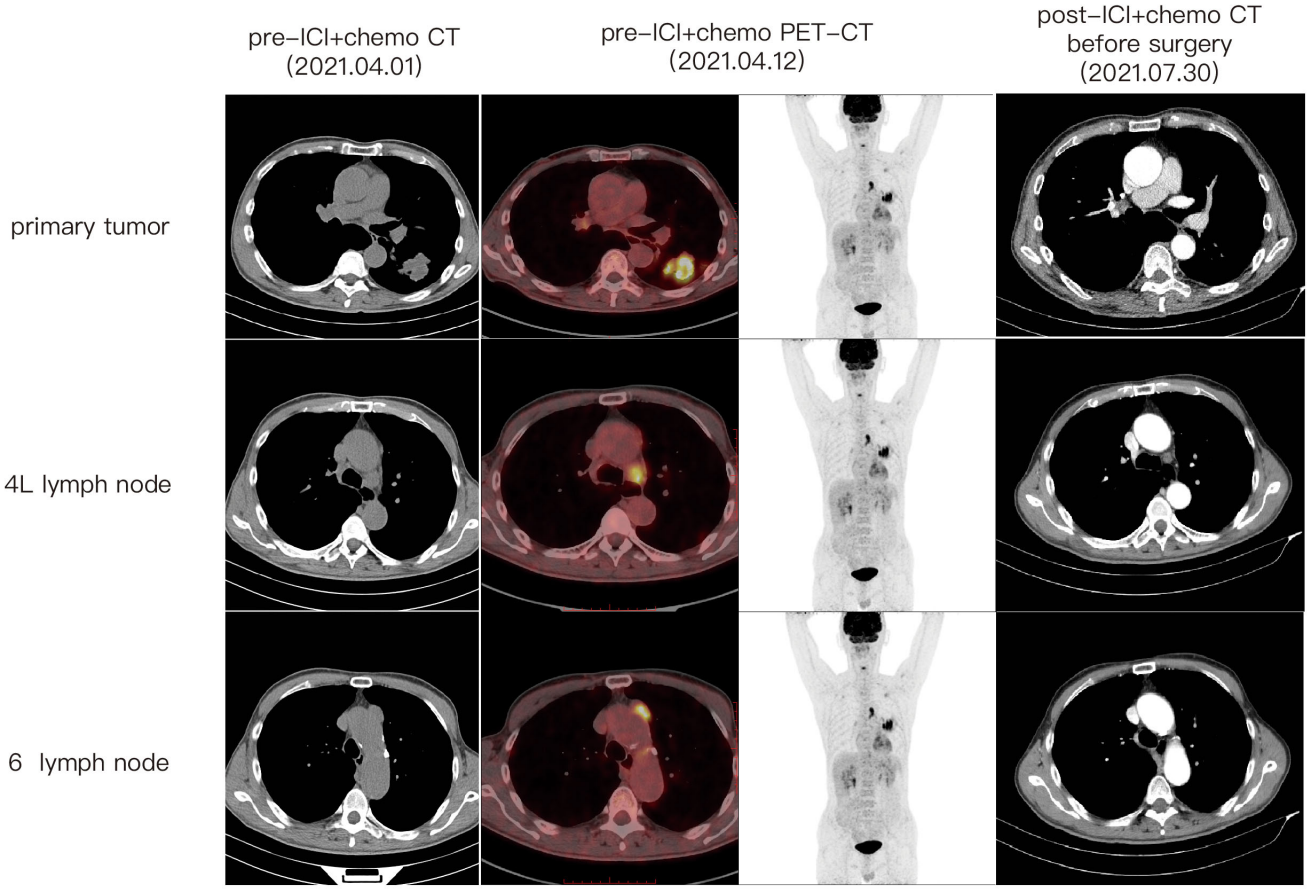
Tumor and lymph nodes shrank significantly after immuno-chemo conversion therapy.

**Figure 2:**
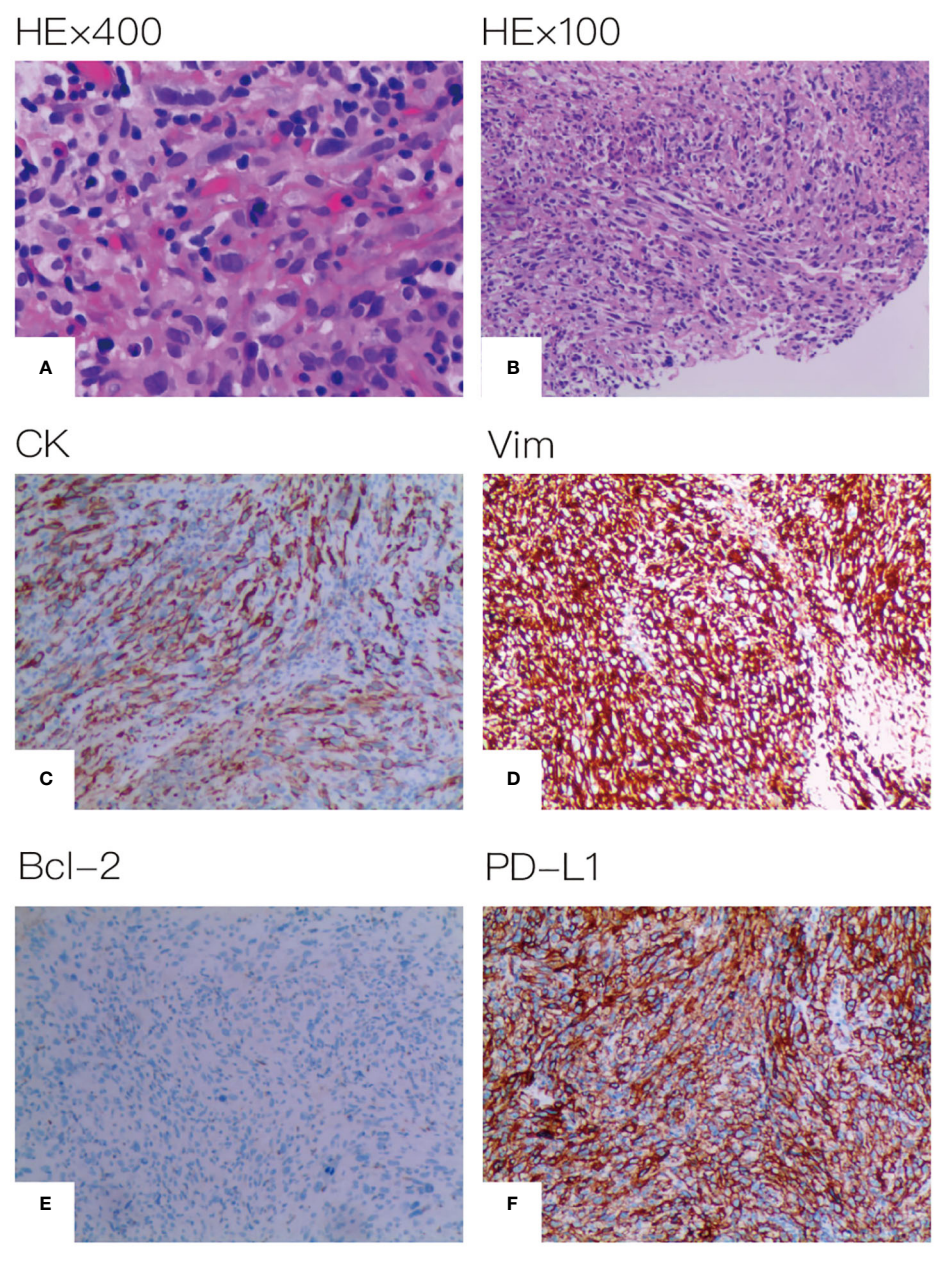
Histopathological assessment of tumor tissue. **(A)** HE×400 **(B)** HE×100 **(C)** CK positive **(D)** Vim positive **(E)** Bcl-2 negative **(F)** PD-L1 expression positive on 95% of tumor cell.

A 67-year-old male patient presented at our hospital, reporting the discovery of a lung mass during his routine annual physical examination. He did not exhibit any noteworthy discomfort or symptoms. In April 2021, the patient was admitted to our hospital and underwent radiographic imaging as well as histopathological examination. Positron-Emission Tomography Computed Tomography (PET-CT) revealed a 40*35 mm mass located in the inferior lobe of the left lung, along with the presence of multiple enlarged lymph nodes in mediastinal regions 4L and 6 (**Figure 1**). These findings were concomitant with heightened 18F-FDG uptake, suggestive of malignancy. No abnormalities were found on enhanced head Magnetic Resonance Imaging (MRI). Bronchoscopic biopsy, in conjunction with immunohistochemical findings, confirmed the diagnosis of pulmonary sarcomatoid carcinoma. However, no biopsy of the enlarged mediastinal lymph nodes was performed. Notably, immunohistochemistry revealed the following results: Cytokeratin (CK) positivity (+), Vimentin (Vim) positivity (+), B-cell lymphoma-2 (Bcl-2) negativity (−), and a high programmed death ligand 1 (PD-L1) tumor proportion score (TPS) of 95% (**Figure 2**). This patient possesses a smoking history spanning over four decades, yet no familial cancer history is evident. Subsequently, the patient received a diagnosis of locally advanced pulmonary sarcomatoid carcinoma (cT2aN2M0, stage IIIA) in alignment with the 8th edition of the American Joint Committee on Cancer (AJCC) TNM classification for lung cancer.

**Figure 3:**
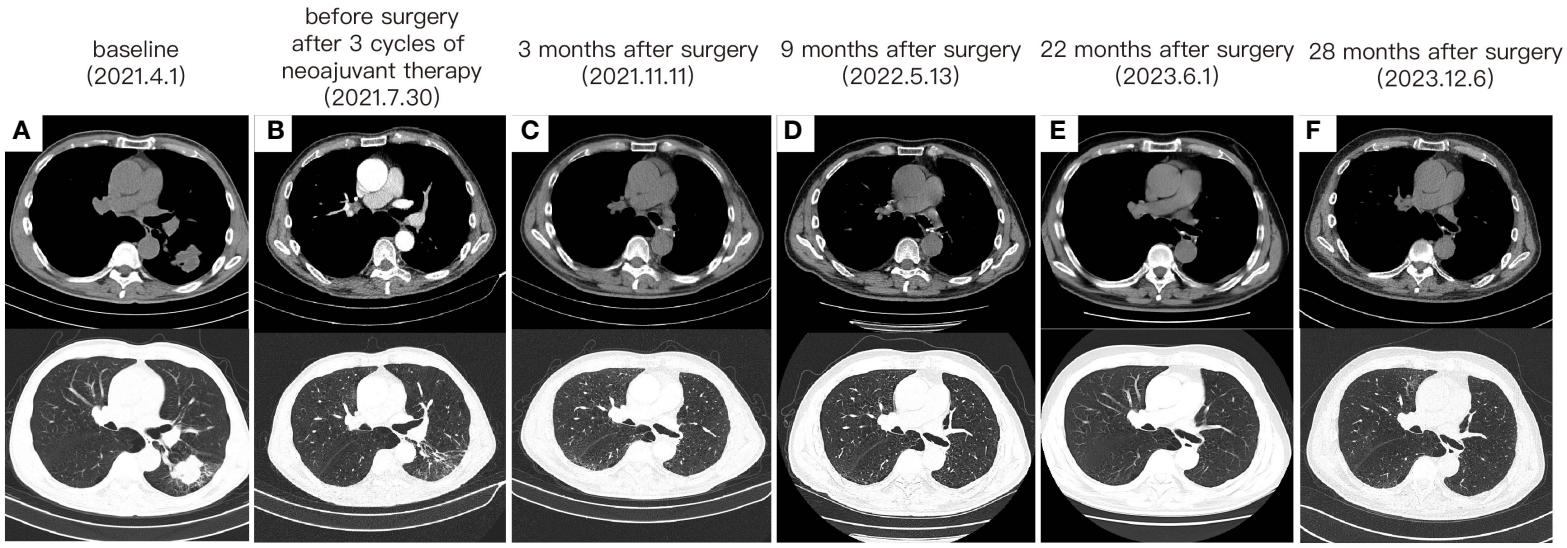
The change of tumor on CT imaging. **(A)** The CT images before any treatment. **(B)** The CT images after 3 cycles of neoadjuvant therapy and before surgery. **(C)** CT taken at 3 months after surgery. **(D)** CT taken at 9 months after surgery. **(E)** CT taken at 22 months after surgery. **(F)** CT taken at 28 months after surgery.

**Figure 4:**
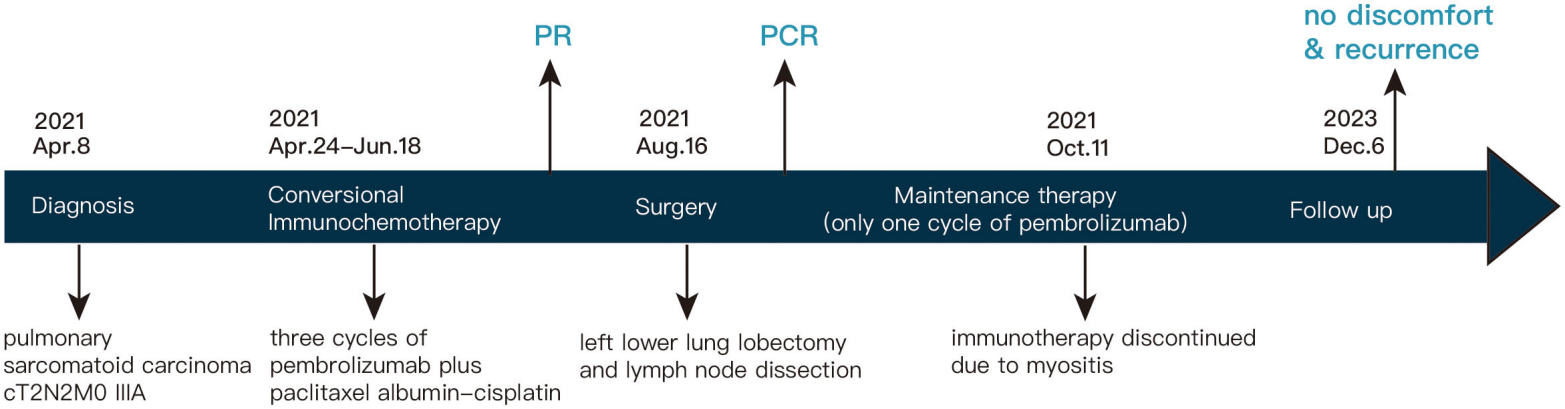
Treatment timeline.

Following a comprehensive evaluation by the Multidisciplinary Team (MDT), it was recommended to initiate chemotherapy plus immunotherapy and to determine the feasibility of surgery at the first efficacy assessment, since the team believed that there was a higher likelihood of tumor regression and conversion to a resectable state following chemotherapy plus immunotherapy. Therefore, the patient received three cycles of paclitaxel-albumin (480mg on day 1) combined with cisplatin (50mg on day 1-2, 40mg on day 3), alongside pembrolizumab (200mg on day 1) by the guidelines for the treatment of non-small cell lung cancer. After this, a contrast-enhanced thoracic CT scan revealed a substantial reduction in both tumor size and lymph nodes (**Figure 1**), yielding a noteworthy achievement of a substantial partial response (PR). After a further MDT discussion, surgical intervention was recommended. Then, the patient underwent left lower lung lobectomy and lymph node dissection. During the surgery, the hilar vessels, bronchi, and lymph nodes were observed to be poorly differentiated and tightly adhered. A scar-like change approximately 1.5 cm in size was noted in the dorsal segment of the left lower lobe, with no tumor visible upon sectioning. Enlargement of lymph nodes was noted in groups 4, 5, 7, 10, and 11, while no enlargement was observed in group 6 lymph nodes. Concurrently, partial adhesions within the thoracic cavity and incomplete pulmonary fissure development were observed, with no evidence of pleural effusion or pleural metastasis. Additionally, the surgery lasted for 4 hours and 45 minutes, with an intraoperative blood loss of approximately 80 milliliters, hence no blood transfusion was necessary. On the second postoperative day, the drainage fluid was clear and the volume was minimal, leading to the removal of the drainage tube. A subsequent chest X-ray revealed a small amount of exudate in the surgical field and pleural effusion in both thoracic cavities. The patient was discharged on the third day following surgery. No postoperative complications were noted. Pathological examination confirmed the absence of viable tumor cells in the tumor specimen and the resected lymph nodes (ypT0N0M0, involving nodes 4, 5, 7, 10, and 11). A PCR was attained. Subsequently, the patient received only one cycle of adjuvant pembrolizumab therapy before the treatment had to be discontinued due to the onset of grade 3 immune-related myositis. The patient exhibited symptoms of muscle weakness and had a muscle strength of grade 4 in all four limbs. This was evidenced by a peak creatine kinase (CK) level of 2250.9 U/L and a peak level of creatine kinase isoenzymes(CK-MB) at 38.3 U/L. Following the cessation of immunotherapy, his myositis experienced a steady improvement without any specific treatment, ultimately leading to complete recovery, but he refused to pursue further adjuvant pembrolizumab therapy upon recovery. He underwent routine reexaminations and follow-up care thereafter. During his most recent follow-up appointment in December 2023, the patient exhibited excellent health and expressed satisfaction with both the treatment options and therapeutic outcomes. CT imaging showed no signs of recurrence, as depicted in **Figure 3**. This culminated in a noteworthy extended EFS exceeding 32 months. The comprehensive timeline of the patient's diagnosis and treatment is depicted in **Figure 4**.

## Discussion

PSC exhibits resistance to conventional therapeutic modalities, culminating in an exceedingly dismal prognosis. In the era of immunotherapy, research findings indicate a promising responsiveness of PSC to ICIs, with the underlying potential mechanism elucidated as follows. Initially, PSC has a pronounced inflammatory cell infiltration, which qualifies it as a “hot tumor”. Secondly, PSC manifests substantial PD-L1 overexpression on tumor cells, accompanied by marked immune infiltration within the tumor microenvironment (TME), notably enriched with CD4+ T lymphocytes and macrophages (4). Thirdly, a significant proportion of PSC cases exhibit an elevated mutational burden (TMB) (> 10 mutations per Mb) (5), which is notably linked to heightened effectiveness in ICI therapy (6). Clinical trials concerning immune checkpoint inhibitors for the treatment of PSC are currently underway (NCT04725448, NCT04888429, NCT04215913). However, most of these investigations and case reports pertaining to advanced or metastatic PSC, with an absence of neoadjuvant/conversional immunochemotherapy reports for PSC thus far. To the best of our knowledge, our case marks the inaugural report of neoadjuvant/conversional immunochemotherapy applied to locally advanced PSC, resulting in the attainment of a pCR and an extended EFS duration. Our case reveals the potential significance of immunochemotherapy within the neoadjuvant/conversional therapy of PSC.

We opted for a preoperative treatment regimen of paclitaxel-albumin plus cisplatin chemotherapy in conjunction with pembrolizumab immunotherapy for the patient, primarily considering that PSC is considered a subset of NSCLC. In the absence of specific treatment guidelines for PSC, our choice was guided by the standard treatments for NSCLC. Postoperatively, the patient received immunotherapy as adjuvant therapy. This model of neoadjuvant therapy-surgery-adjuvant therapy is now a popular trend in the current exploration of novel NSCLC treatment strategies. At the 2023 ASCO Annual Meeting, the KEYNOTE-671 trial, a large-scale, randomized, double-blind Phase III clinical study, presented its results. The study aimed to evaluate the efficacy and safety of pembrolizumab combined with chemotherapy as neoadjuvant therapy for patients with resectable Stage II, IIIA, and IIIB NSCLC, followed by pembrolizumab monotherapy as adjuvant therapy. The findings indicated that, among patients with resectable Stage II-IIIB (N2) NSCLC, pembrolizumab perioperative therapy significantly improved patients’ EFS, pCR, and major pathological response (mPR) compared to those who received neoadjuvant chemotherapy and surgery alone (7).

Our PSC case has an excellent efficacy to conversional immunochemotherapy. Multiple factors underpin this phenomenon. First, the patient has a high PD-L1 expression (TPS 95%), which is usually associated with better efficacy of ICIs. Nonetheless, a retrospective investigation by Inomata et al. on ICI treatment of PSC revealed that SC patients with high PD-L1 expression had no link between tumor PD-L1 expression level and survival time, and even some individuals with PD-L1 TPS≥50% had early progression or death (8). These studies exhibited considerable heterogeneity in their findings, potentially attributable to the limited sample size. Second, our case manifested immune-associated myositis, a potential sign of notable therapeutic effectiveness. An array of retrospective and prospective investigations has consistently demonstrated that patients encountering immune-related adverse events (irAEs) exhibit superior Progression-Free Survival (PFS), OS, Objective Response Rate (ORR), and Disease Control Rate (DCR) when subjected to ICIs for NSCLC in comparison to their irAE-absent counterparts (9–11). Our case is consistent with these studies.

Presently, the EFS in our case extends beyond the two-year mark. This long EFS may be since he reached PCR after conversional therapy. PCR is an earlier predictor of longer EFS. The Checkmate 816 study (12) unveiled a robust correlation between EFS and PCR, with EFS being longer in PCR patients than non-PCR patients. Greater EFS benefit was seen in the PD-L1 expression ≥1% subgroup compared to the PD-L1 expression <1% group. Our case has a high PD-L1 expression and achieved PCR, this may be a reason for his long EFS.

Intriguingly, our case has remained free from relapse for nearly two years following the cessation of immunotherapy. His EFS achieved longer than 26 months and the patient’s current state of health is favorable. As we know, the median OS was only 16.9 months for stage I-II patients and 5.8 months for operable stage III PSC patients before the advent of ICIs (13). Comparatively, it is indeed quite astonishing that our case can have such a long EFS. It merits exploration whether the continuation of immunotherapy is requisite in cases where a PCR has been attained.

This case has certain limitations. First, he did not undergo gene mutation testing due to the limited biopsy specimen. It is reported that PSC has a much higher mutation rate of METex14 skipping than other types of NSCLC, which can reach 22% to 31.8% (14, 15). However, several retrospective studies showed that NSCLC patients with MET mutations had limited benefit from immunotherapy (16–19). According to this speculation, the present case may not exhibit METex14 skipping. Second, because to the restricted specimen size, we were unable to detect the patient’s TME and investigate the likely mechanism of considerable efficacy. Despite the case’s limitations, its contribution to the neoadjuvant/conversional field of PSC cannot be denied.

## Conclusion

According to our knowledge, this is the first example of effective conversional immunochemotherapy in locally advanced PSC, in which the patient achieved PCR and did not relapse for over two years after immunotherapy was discontinued. It presents a viable option for those with comparable PSC.

## Data availability statement

The original contributions presented in the study are included in the article/supplementary material. Further inquiries can be directed to the corresponding authors.

## Ethics statement

Written informed consent was obtained from the individual(s) for the publication of any potentially identifiable images or data included in this article.

## Author contributions

YG: Data curation, Formal analysis, Investigation, Visualization, Writing – original draft, Writing – review & editing, Software. XL: Supervision, Writing – review & editing, Formal analysis, Resources. TH: Writing – review & editing, Formal analysis, Writing – original draft. ZQ: Writing – original draft, Resources. FM: Writing – review & editing, Investigation. AH: Writing – original draft, Visualization. CL: Conceptualization, Data curation, Formal analysis, Resources, Supervision, Writing – original draft, Writing – review & editing. YW: Resources, Software, Supervision, Writing – review & editing.
